# Effect of Statins on Renal Function in Chronic Kidney Disease Patients

**DOI:** 10.1038/s41598-018-34632-z

**Published:** 2018-11-02

**Authors:** Ping-Jen Hu, Mei-Yi Wu, Tsu-Chen Lin, Tzu-Ting Chen, Yun-Chun Wu, Sui-Lung Su, Kuo-Cheng Lu, Jin-Shuen Chen, Fung-Chang Sung, Chien-Te Lee, Yu Yang, Shang-Jyh Hwang, Ming-Cheng Wang, Yung-Ho Hsu, Hung-Yi Chiou, Chung-Shun Wong, Yuh-Feng Lin

**Affiliations:** 1 Division of Gastroenterology, Taipei Medical University-Shuang Ho Hospital, Taipei, Taiwan; 2 Department of Nephrology, Taipei Medical University-Shuang Ho Hospital, Taipei, Taiwan; 3 Department of Internal Medicine, School of Medicine, College of Medicine, Taipei Medical University, Taipei, Taiwan; 4 Institute of Epidemiology and Preventive Medicine, College of Public Health, National Taiwan University, Taipei, Taiwan; 5 School of Medicine, College of Medicine, Taipei Medical University, Taipei, Taiwan; 6 School of Public Health, National Defense Medical Center, Taipei, Taiwan; 7 Division of Nephrology, Department of Medicine, Fu-Jen Catholic University Hospital, School of Medicine, Fu-Jen Catholic University, Taipei, Taiwan; 8 Division of Nephrology, Department of Medicine, Tri-Service General Hospital, National Defense Medical Center, Taipei, Taiwan; 9 School of Public Health, Graduate Institute of Clinical Medical Science, China Medical University, Taichung, Taiwan; 10 Division of Nephrology, Kaohsiung Chang Gung Memorial Hospital, Chang Gung Medical University, Kaohsiung, Taiwan; 11 The Division of Nephrology, Changhua Christian Hospital, Changhua, Taiwan; 12 Division of Nephrology, Department of Medicine, Kaohsiung Medical University Hospital, Kaohsiung, Taiwan; 13 Division of Nephrology, Department of Internal Medicine, Cheng Kung University Medical Center, Tainan, Taiwan; 14 School of Public Health, College of Public Health and Nutrition, Taipei Medical University, Taipei, Taiwan; 15 Graduate Institute of Clinical Medicine, College of Medicine, Taipei Medical University, Taipei, Taiwan; 16 Department of Emergency Medicine, Taipei Medical University-Shuang Ho Hospital, Taipei, Taiwan; 17 Department of Emergency Medicine, School of Medicine, College of Medicine, Taipei Medical University, Taipei, Taiwan

## Abstract

Dyslipidemia is associated with glomerular injury. However, the effect of statins on chronic kidney disease (CKD) progression remains controversial. We aimed to investigate the efficacy of statins for renal protection in patients with CKD. The retrospective cohort study comprised 3441 patients diagnosed with CKD in multiple medical centers. We divided the patients into two cohorts based on statin prescription, and compared proportions and risks of CKD progression events between the two groups. CKD progression event was defined as an average annual decline of eGFR >5 mL/min/1.73 m^2^ or advancement to the dialysis stage. The result revealed that among all incident patients with CKD, 28.7% and 30.3% of the users and nonusers demonstrated CKD progression, respectively. The crude odds ratio (OR) of CKD progression was 0.93 [95% confidence interval (CI) 0.78–1.10]. After adjustment for baseline characteristics, the adjusted OR was 0.80 (95% CI 0.63–1.01). The sensitivity analysis results showed consistent OR for CKD progression, stratification by age, sex, Charlson score, and statins use within 1 year before index date. The effect of statins was significant in patients with CKD stage 3B-5 (OR 0.68, 95% CI 0.48–0.95), but not statistically significant in those with CKD stage 1–3A (OR 0.97, 95% CI 0.68–1.38). The effect of statins was significant in patients with proteinuria ≥1000 mg/day (OR 0.63, 95% CI 0.43–0.92), but not statistically significant in those with proteinuria <1000 mg/day (OR 1.02, 95% CI 0.74–1.41).

## Introduction

Chronic kidney disease (CKD) is a global health concern^1^. According to systemic review data and a meta-analysis of observational studies until September 2014, the global prevalence of CKD was approximately 13.4%, and the prevalence of stage 3–5 CKD was 10.6%^1^. An estimated 5.4 million people will be receiving dialysis due to end-stage renal disease (ESRD) in 2030, and globally, the number of patients receiving dialysis is increasing the fastest in Asia^2^. According to the 2017 United States Renal Data System (USRDS) report, the highest prevalence and incidence of ESRD among all countries investigated was noted in Taiwan^3^. Among patients with ESRD in Taiwan, median estimated glomerular filtration rate (eGFR) at initial dialysis was approximately 4.7 mL/min/1.73 m^2^ ^4^.

Cardiovascular disease (CVD), which may occur even at the earliest stages of CKD without manifestations of vascular disease, is the leading cause of morbidity and mortality among patients with CKD^5^. CKD is associated with increased CVD risk, severity of which increases as kidney function deteriorates^6^. Statins are the mainstay of primary and secondary prevention of CVD in the general population^7^. The Kidney Disease Improving Global Outcomes (KDIGO) lipid management guidelines suggest statin initiation for primary prevention in all patients with CKD above the age of 50 years, and all adult CKD patients with diabetes who are not receiving dialysis^8^. In Taiwan, in adults with eGFR <60 mL/min/1.73 m^2^ and without chronic dialysis, statins therapy is recommended only if low-density lipoprotein cholesterol levels are ≥100 mg/dL^9^.

Patients with CKD are more likely to exhibit elevated triglyceride and low high-density lipoprotein cholesterol levels, which were the risk factors for CVD^10^. On the basis of the experimental evidence, dyslipidemia is associated with tubulointerstitial and glomerular injuries, which may result in glomerulosclerosis^11,12^. Statins inhibit HMG-CoA reductase activity and thus play a beneficial role in dyslipidemia treatment^13^. Many studies have investigated the renoprotective effects of statins. The ALERT trial and Scandinavian Simvastatin Survival Study have demonstrated that statins slowed CKD progression^14,15^; however, other studies such as the SHARP study, ALLHAT study, and ASUCA trial have reported that statins exhibit little to no effects on CKD progression^16–18^. Recent systematic reviews and meta-analyses have indicated that statins do not reduce risk of kidney failure events in adults not receiving dialysis, where kidney failure events are defined as 25–50% decrease in eGFR, doubling of serum creatinine level, or advancement to ESRD stage during follow-up period; however, according to the same reports, statins may reduce proteinuria and eGFR decline^19^. Therefore, we evaluated the renoprotection efficacy of statins in patients with CKD.

## Methods

### National Health Insurance Database and Multicenter CKD Cohort

The National Health Insurance Database (NHID) is a research database developed by the Ministry of Health and Welfare^20^, including National Health Insurance claims data more than 99% of 23 million Taiwan residents. The Ministry of Health and Welfare annually releases the Longitudinal Health Insurance Database for research purposes, which contains the insured population’s registration files and medical claims data, including data on basic demographic characteristics, inpatient and ambulatory care, diagnostic codes, medical expenditure, operations, prescriptions, examinations, and procedures. From January 1, 2008, to Dec 31, 2013, a multicenter project was conducted to survey risk factors for CKD in the Taiwan population. This project was supported by the Bureau of Health Promotion, Ministry of Health and Welfare, Taiwan. The present CKD retrospective observational cohort study is based on the data of 7956 patients from the multicenter project. We used national identification numbers to link laboratory data from study hospitals to the NHID. This research project was approved by the Ethics Committee of Taipei Medical University-Shuang Ho Hospital (TMU-JIRB 20124036), Tri-Service General Hospital (TSGHIRB 100-05-197), Cardinal Tien Hospital (TMU-JIRB 201204035), Changhua Christian Hospital (CCHIRB 20405), Kaohsiung Medical University Chung-Ho Memorial Hospital (KMUHIRB 20120019), Kaohsiung Chang Gung Memorial Hospital (101-1096B), National Cheng Kung University Hospital (A-ER-101-117) and China Medical University Hospital (DMR101-IRB2-273(CR-1)). After a complete explanation of the study, written informed consent was obtained from all participants. All clinical and biological samples were collected after patient consent. All the study methods were in accordance with the guidelines approved by the joint institutional review board and aforementioned governmental regulations.

### Study Population

**Figure 1:**
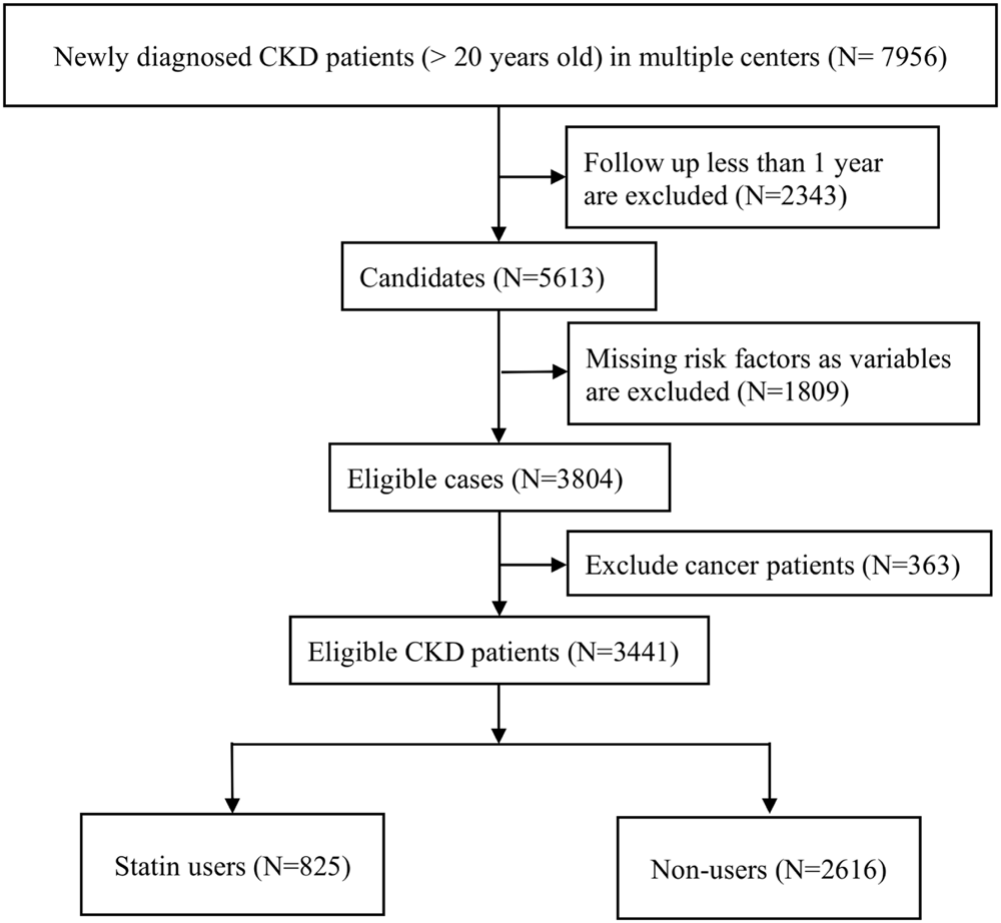
Flowchart demonstrates the selection criteria and process of eligible CKD patients.

The initial study cohort comprised 7956 patients diagnosed with CKD between November 1, 2008, and June 30, 2013, in multiple medical centers (Fig. 1). We calculated eGFRs by using the Chronic Kidney Disease Epidemiology Collaboration equation, as recommended by KDIGO guidelines. The inclusion criteria were as follows: kidney damage evident as structural or functional abnormalities or eGFR of <60 mL/min/1.73 min^2^ for more than 3 months, with this eGFR being used to determine CKD stage^21^. The exclusion criteria were as follows: age <20 years, follow-up duration <1 year, absence of major variables [e.g., age; sex; baseline CKD stage; and Charlson comorbidity scores, including these for diabetes mellitus (DM), coronary artery disease, and stroke], and cancer patients. The multiple imputation method was used to account for missing data on smoking, alcohol use, and betel nut use^22^. We input missing data on the basis of patients’ sex and CKD stage. After patients with loss to follow-up, with missing data, or with cancer were removed from the database, the final study cohort comprised 3441 CKD patients.

### Study Design

The patients were divided into two cohorts based on statin prescription: statin users (n = 825, those receiving a ≥90-day statin prescription within 180 days of the index date) and nonusers (n = 2616, those not receiving statins or receiving <90-day statin prescription within 180 days of the index date). Patients were individually tracked from the index date to December, 2015, to determine CKD progression. The primary outcome was CKD progression, defined as an average annual decline of eGFR >5 mL/min/1.73 m^2 ^^23^ or advancement to the dialysis stage.

### Statistical Analysis

We compared the demographic data between statin users and nonusers by using Pearson’s chi-squared test and *t* test. We calculated proportions of CKD progression events; then, a logistic regression model was used to calculate crude odds ratios (ORs) between statin users and nonusers. After additional adjustments for the potential confounding factors including age, sex, comorbidities, smoking, alcohol use, betel nut use, body mass index (BMI), baseline eGFR, baseline urine protein-to-creatinine ratio (UPCR), and statin use within 1 year before the index date, adjusted ORs (aORs) were calculated. We also performed sensitivity analysis after stratification according to age (<65 and ≥65 years), sex, Charlson comorbidity index score (≤3 and >3), baseline CKD stage (1–3 A and 3B–5), proteinuria (<1000 mg/day and ≥1000 mg/day) and stain use within 1 year before the index time. All analyses were performed the SAS system for Windows (version 9.3.1; SAS Institute Inc., Cary, NC, USA). *P* < 0.05 was considered significant.

## Results

### Demographic Characteristics

From November 1, 2008, to June 30, 2013, across multiple medical centers, a total of 7956 patients with CKD, aged 20–85 years, fulfilling the inclusion criteria were identified. Among them, those who had ever received dialysis or kidney transplant were excluded. After the additional exclusion of patients with a follow-up of <1 year (n = 2343), with incomplete or missing data (n = 1809), and with cancer (n = 363), 3441 patients with CKD were enrolled (Fig. 1). Detailed demographic information for the cohort is provided in Table 1. The mean age of statin users and nonusers were 62.99 ± 12.82 and 62.05 ± 14.62 years, respectively; 52.73% and 57.72% of statin users and nonusers were men, respectively. Among all patients, 1717 and 1724 exhibited early (1–3A)- and late (3B–5)-stage CKD, respectively. Compared with the nonusers, a higher proportion of the users had DM (53.45% vs 39.60%), hypertension (84.61% vs 76.26%), coronary artery disease (3.39% vs 2.60%), stroke (18.18% vs 16.59%), BMI of >25 (57.09 vs 44.38%), and more statin use within 1 year before the index date (86.79% vs 14.33%). The mean eGFR of the users and nonusers was 52.58 ± 34.21 and 51.25 ± 36.37 mL/min/1.73 m^2^, respectively. Furthermore, the users had a significantly higher baseline cholesterol, fasting glucose and HbA1c levels than did the nonusers (p = 0.0023; p = 0.0016; p = 0.003, respectively). Baseline levels of serum albumin, electrolytes, uric acid, hemoglobin, hematocrit, and proteinuria did not differ significantly between the groups. The following levels of cholesterol of the users and nonusers was 177.6 ± 43.15 and 177.14 ± 41.06 mg/dl, respectively (p = 0.8049).

**Table 1:** Baseline Characteristics of Patients with CKD Stage 1–5.

Characteristic	Statin Users	Statin Nonusers	*P-value*
Number of patients	825	2616	
Age, mean (SD), years	62.99 ± 12.82	62.05 ± 14.62	0.0755
Age group, years			<0.0001
20–44	74 (8.97)	340 (13.00)	
45–64	355 (43.03)	1057 (40.41)	
65–74	252 (30.55)	658 (25.15)	
≥75	144 (17.45)	561 (21.44)	
Male	435 (52.73)	1510 (57.72)	0.0116
Waist, cm	89.18 ± 11.56	86.47 ± 11.57	<0.0001
Body mass index, kg/m^2^			<0.0001
<18.5	11 (1.33)	87 (3.33)	
18.5–24.9	343 (41.58)	1368 (52.29)	
25–29.9	338 (40.97)	870 (33.26)	
≥30	133 (16.12)	291 (11.12)	
Smoking	203 (24.61)	659 (25.19)	0.7352
Alcohol	83 (10.06)	268 (10.24)	0.8790
Betel nut	44 (5.33)	155 (5.93)	0.5255
Statin drug used within 1 year before the index date	716 (86.79)	375 (14.33)	<0.0001
Baseline CKD stage			0.3652
1–3A	423 (51.27)	1294 (49.46)	
3B–5	402 (48.73)	1322 (50.54)	
Comorbidities before the index date			
Diabetes Mellitus	441 (53.45)	1036 (39.60)	<0.0001
Coronary artery disease	28 (3.39)	68 (2.60)	0.2269
Stroke	150 (18.18)	434 (16.59)	0.2883
Hypertension	698 (84.61)	1995 (76.26)	<0.0001
Charlson comorbidity index			0.0034
≤3	443 (53.70)	1567 (59.90)	
4–5	222 (26.91)	645 (24.66)	
>5	160 (19.39)	404 (15.44)	
Charlson comorbidity index, mean (SD)	3.63 ± 2.14	3.3 ± 2.17	0.0002
Baseline examination data			
Fasting glucose, mg/dL	120.44 ± 44.07	114.32 ± 43.06	0.0016
HbA1c, %	7.16 ± 1.56	6.87 ± 2.10	0.0030
Total cholesterol, mg/dL	188.27 ± 48.45	182.18 ± 44.01	0.0023
Triglyceride, mg/dL	146.71 ± 85.41	139.28 ± 105.12	0.0516
Albumin, g/dL	4.05 ± 0.90	4.38 ± 11.53	0.2829
Serum Na, mmol/L	141.99 ± 55.3	139.34 ± 5.77	0.3012
Serum K, mmol/L	4.75 ± 5.94	4.52 ± 3.34	0.3700
Serum Ca, mg/dL	9.04 ± 0.63	8.98 ± 2.07	0.2995
Serum P, mg/dL	3.99 ± 1.68	4.01 ± 1.02	0.8228
Uric acid, mg/dL	7.01 ± 2.66	6.97 ± 2.49	0.6780
Hb, mg/dL	12.22 ± 2.27	12.10 ± 2.54	0.2592
Hct, mg/dL	36.79 ± 18.55	35.85 ± 6.69	0.1864
UPCR, mg/g	5682.23 ± 124358.24	1063.28 ± 2758.89	0.2864
eGFR, mL/min/1.73 m^2^	52.58 ± 34.21	51.25 ± 36.37	0.3383
Follow-up examination data			
Fasting glucose, mg/dL	118.24 ± 39.48	114.75 ± 40.31	0.0604
HbA1c, %	6.83 ± 1.35	6.65 ± 1.38	0.0219
Total cholesterol, mg/dL	177.60 ± 43.15	177.14 ± 41.06	0.8049
Triglyceride, mg/dL	143.19 ± 92.53	138.64 ± 123.98	0.3159
Albumin, g/dL	4.03 ± 0.50	4.72 ± 23.90	0.2826
Serum Na, mmol/L	138.33 ± 9.21	138.48 ± 6.98	0.7455
Serum K, mmol/L	4.40 ± 0.64	4.43 ± 1.34	0.4016
Serum Ca, mg/dL	9.06 ± 0.86	9.03 ± 1.14	0.5658
Serum P, mg/dL	4.20 ± 1.22	4.13 ± 1.24	0.2760
Uric acid, mg/dL	6.95 ± 4.21	6.80 ± 3.44	0.4404
Hb, mg/dL	12.01 ± 2.48	12.51 ± 20.62	0.3069
Hct, mg/dL	35.70 ± 6.48	36.15 ± 20.90	0.4200
UPCR, mg/g	1315.04 ± 2029.19	1225.66 ± 2219.41	0.4392
eGFR, mL/min/1.73 m^2^	49.07 ± 33.47	52.00 ± 41.07	0.0487

SD, standard deviation; CKD, chronic kidney disease; UPCR, urine protein and creatinine ratio; eGFR, estimated glomerular filtration rate.

### Long-Term Risks of CKD Progression Events

Table 2 presents proportions and risk of CKD progression events. CKD progression occurred in 237 (28.73%) users and 793 (30.31%) nonusers. The crude OR and aOR (after adjustment for confounding factors) for CKD progression were 0.93(95% confidence interval [CI], 0.78–1.10), and 0.80 (95% CI, 0.63–1.10), respectively. Stratified by CKD stage, the statin effects in reduction of CKD progression was significant in CKD stage 3B-5 (aOR 0.68, 95% CI 0.48–0.95), but not statistically significant in those with CKD stage 1–3A (aOR 0.97, 95% CI 0.68–1.38).

**Table 2:** Proportion and risk of CKD progression in patients with CKD stage 1–5, according to stain use.

	No. of events	No. of patients	Proportion (%)	Unadjusted odds ratio	Adjusted odds ratio^b^
Total					
Statin Users	237	825	28.73	0.93 (0.78, 1.10)	0.80 (0.63, 1.01)
Statin Nonusers	793	2616	30.31	1.00	1.00
Stage 1–3a					
Statin Users	121	423	28.61	1.08 (0.85, 1.38)	0.97 (0.68, 1.39)
Statin Nonusers	350	1294	27.05	1.00	1.00
Stage 3b-5					
Statin Users	116	402	28.86	0.81 (0.63, 1.03)	0.68 ^a^ (0.48, 0.95)
Statin Nonusers	443	1322	33.51	1.00	1.00

Note: Values in parentheses are 95% confidence interval.

^a^P < 0.001.

^b^Adjustments were made for age, sex, diabetes mellitus, coronary artery disease, stroke, cancer, Charlson comorbidity index, Statin drug used within 1 year before the index date, body mass index, smoking, alcohol, betel nut, urine protein and creatinine ratio, and baseline estimated glomerular filtration rate.

**Figure 2:**
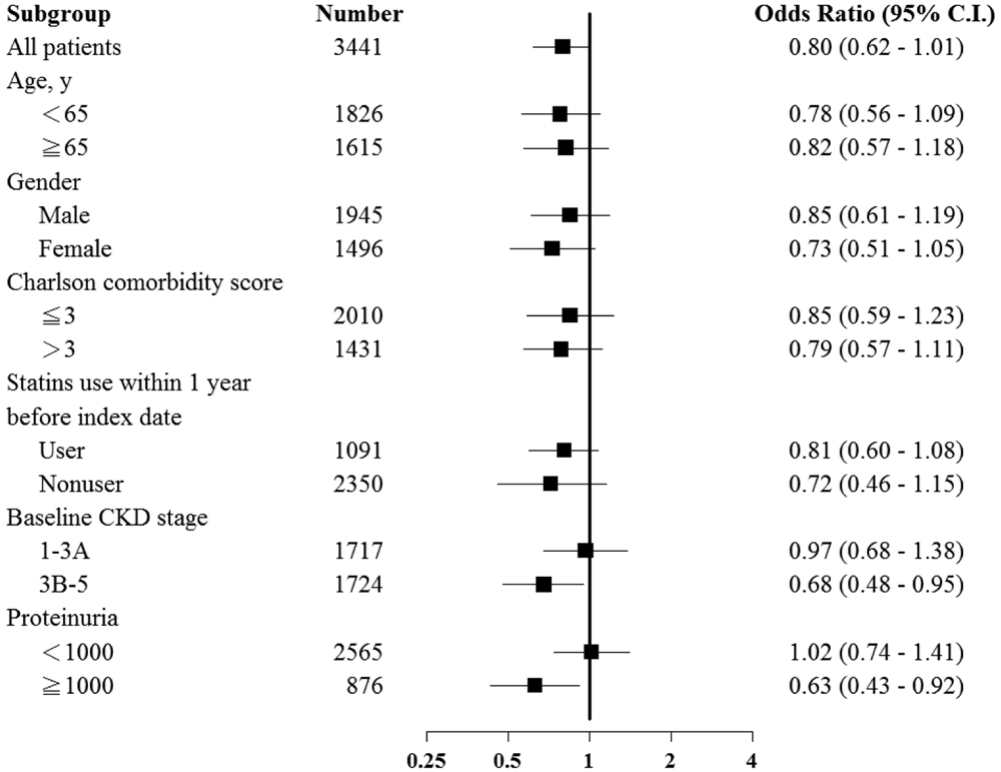
Subgroup analysis of risk of CKD among population with statin users and non-users.

Figure 2 presents the sensitivity analysis results. Consistent aORs were noted for CKD progression, stratification by age, sex, Charlson score, and statins use within 1 year before index date. The effect of statins was significant in patients with proteinuria ≥1000 mg/day (aOR 0.63, 95% CI 0.43–0.92), but not statistically significant in those with proteinuria <1000 mg/day (aOR 1.02, 95% CI 0.74–1.41).

## Discussion

Our study demonstrated several critical findings with potential implications in therapies for CKD. In CKD stage 3B-5, statin use was significantly associated with a decrease in the number of CKD progression events, defined as an annual eGFR decline of ≥5 or advancement to the dialysis stage. The renoprotective effect of statins had the trend but not statistically significant in all CKD patients after adjustment for demographic and clinical characteristics including age, sex, comorbidities, alcohol use, betel nut use, smoking, BMI, baseline eGFR, baseline UPCR, and statin use within 1 year before the index date (crude OR 0.93, 95% CI 0.78–1.10; aOR 0.80, 95% CI 0.62–1.01). In the subgroup of patients with CKD stage 3B-5 (aOR 0.68, 95% CI 0.48–0.95), and those with proteinuria ≥1000 mg/day (aOR 0.63, 95% CI 0.43–0.92), statins exhibited renoprotective effects with statistically significant.

Because of their main mechanism of inhibiting HMG-CoA reductase activity, statins are widely used to treat hyperlipidemia. In addition, statins have other therapeutic benefits, such as anti-inflammatory and antioxidant properties, apoptosis induction, vascular smooth muscle proliferation inhibition, platelet activation and aggregation reduction, and increase in atherosclerotic plaque stability^24^. Many of these effects are potentially arise from small G-protein disruption^24^. Because of its resultant biological and genetic stress, the activation of G-protein signaling is pivotal in renal pathologies^25^.

The effects of statins on CKD progression remain subject to debate. A 2016 meta-analysis^19^ examined the data of 57 randomized control trials (RCTs), with 143,888 participants and 8,498 kidney failure events and suggested that statin therapy results in mild decreases in proteinuria levels and an eGFR decline of 0.41 mL/min/1.73 m^2^ per year. Su *et al*.^19^ defined kidney failure events as 25–50% decrease in eGFR, doubling of serum creatinine level, or advancement to ESRD stage during the follow-up period. Statin use did not prevent or mitigate these kidney failure events, consistent with the results of a systemic review conducted by Zhong *et al*.^26^; in total, 23 RCTs with 39,419 participants were analyzed, and the authors concluded that statins significantly reduced microalbuminuria, proteinuria, and clinical deaths, but did not slow the clinical progression of non-end-stage CKD significantly.

The 2012 KDIGO guidelines^27,28^ suggest that dialysis should be initiated when the eGFR is approximately 5–9 mL/min/1.73 m^2^. The 2014 Canadian Society of Nephrology clinical practice guidelines suggest that chronic dialysis should be initiated when eGFR decreases to 6 mL/min/1.73 m^2^, even if no clinical indicators are exhibited. Although data have shown high prevalence and incidence of ESRD in the Taiwan population^3^, patients in Taiwan begin dialysis with poor clinical conditions (mean hematocrit 24.2%, mean serum albumin 3.2 g/dL) with low residual renal function (eGFR 4.7 mL/min/1.73 m^2^)^4^. Hwang *et al*.^4^ attributed the delay in dialysis to patient awareness and attitudes toward dialysis treatment; in Taiwan, to initiate dialysis, pre-ESRD patients tend to wait for the presentation of uremic symptoms and other clinical signs, rather than following the eGFR criteria. The effects of dialysis initiation timing remains controversial. Although dialysis initiation timing is relatively late in Taiwan, mortality due to CKD is low^4^.

Our study enrolled patients from multiple medical centers in Taiwan, and national identification numbers were used to link patients with corresponding data in the NHID^20^. In contrast to definitions used in previous studies, we identified CKD progression events as either an annual average eGFR decline >5 mL/min/1.73 m^2^ or advancement to the dialysis stage. Because observational studies are prone to bias and being confounded, sensitivity analyses were performed, the results of which suggested that statin use benefited in those with CKD stage 3B-5 and proteinuria ≥1000 mg/day. A possible explanation for this finding is that patients may require a higher dosage of statin therapy. Shepherd *et al*.^29^ reported that although some eGFR improvement occurred after low-dosage (10 mg/day) atorvastatin treatment, high-dosage atorvastatin led to a more significant eGFR improvement. However, a 2014 meta-analysis^30^ involving 6 RCTs compared high-intensity statin therapy (atorvastatin 80 mg or rosuvastatin 20 or 40 mg) with moderate-to-mild statin treatment or placebo and noted that the effect of the high-intensity and moderate-to-mild therapies on eGFR improvement was not substantially different; regarding the safety of statin use, the prevalence of adverse events was low, and the pooled results showed no significant differences in adverse event prevalence among patients receiving high-intensity or nonintensive statin therapy or placebo^30^. Therefore, the effect of high-intensity statin on renal function is difficult to determine conclusively, and more evidence from high-quality studies is required.

This study has some limitations. First, this was a retrospective cohort study with a short follow-up period (1–6 years). Second, our criterion for defining CKD progression (annual eGFR decline >5 mL/min/1.73 m^2^) was broader than that of similar studies. It is worth noting that the beneficial effects of statin can be seen in patients with more than one gram of urine protein, because urinary protein itself is an indicator that can lead to deterioration of kidney function.

## Conclusion

This national cohort study on CKD found that statins effectively delay CKD progression in CKD stage 3B-5 patients, particularly among those with proteinuria ≥1000 mg/day, for whom the benefits of treatment are clear. Thus, statin therapy may have a net clinical benefit for preventing CKD progression, particularly considering the high burden of dialysis. The protective effect of CKD on kidneys may differ according to statin dosage, and additional evidence is required to confirm these benefits. Our results suggest that statin usage in CKD with stage 3B-5 and those with proteinuria ≥1000 mg/day is critical, particularly for targeting CKD progression outcomes.
